# Metabolic Dysfunction-Associated Steatotic Liver Disease and Incretin Receptor Agonists: A Metabolic Approach to Halting Liver Disease Progression

**DOI:** 10.3390/medicina62050986

**Published:** 2026-05-18

**Authors:** Ludovico Abenavoli, Anna Giulia Loricchio, Ivo Lopez, Domenico Morano, Abdulrahman Ismaiel, Dan Lucian Dumitrascu, Francesco Luzza

**Affiliations:** 1 Department of Health Sciences, University of Catanzaro “Magna Graecia”, 88100 Catanzaro, Italy; annagiulia.loricchio@studenti.unicz.it (A.G.L.); ivo.lopez@studenti.unicz.it (I.L.); domenico.morano1@studenti.unicz.it (D.M.); luzza@unicz.it (F.L.); 2 Center for Chronic Liver Diseases, “Renato Dulbecco” University Hospital, 88100 Catanzaro, Italy; 3 2nd Department of Internal Medicine, “Iuliu Hatieganu” University of Medicine and Pharmacy, 400006 Cluj-Napoca, Romania; ismaiel.abdulrahman@umfcluj.ro (A.I.); ddumitrascu@umfcluj.ro (D.L.D.)

**Keywords:** treatment, chronic liver disease, management, liver steatosis

## Abstract

Metabolic dysfunction-associated steatotic liver disease (MASLD) is strongly associated with metabolic abnormalities, shares pathophysiological pathways with metabolic syndrome, and has become a leading cause of chronic liver disease in industrialized nations. In the absence of approved pharmacological treatments and due to its high risk of progression to advanced fibrosis, MASLD represents a significant clinical challenge. Incretin-based therapies, originally developed for the treatment of type 2 diabetes mellitus and obesity, have recently gained attention as promising therapeutic strategies in hepatology. Among them, GLP-1 receptor agonists have shown efficacy in reducing hepatic steatosis, inflammation, and fibrosis-related biomarkers, primarily through weight loss and enhanced insulin sensitivity. Dual agonists targeting both GLP-1 and GIP receptors, such as tirzepatide, have demonstrated superior outcomes in improving hepatic and metabolic parameters. Emerging agents like cotadutide (a GLP-1/glucagon receptor agonist) and retatrutide (a GLP-1/GIP/glucagon triagonist) represent a novel therapeutic frontier, with early clinical data indicating potent hepatoprotective effects and favorable metabolic remodeling. This narrative review examines the hepatoprotective potential of incretin-based therapies, highlighting how targeted intervention on the underlying metabolic dysfunction may lead to significant improvements in MASLD. These therapies may also exert beneficial effects on fibrosis progression; however, the currently available evidence remains limited.

## 1. Introduction

**Figure 1:**
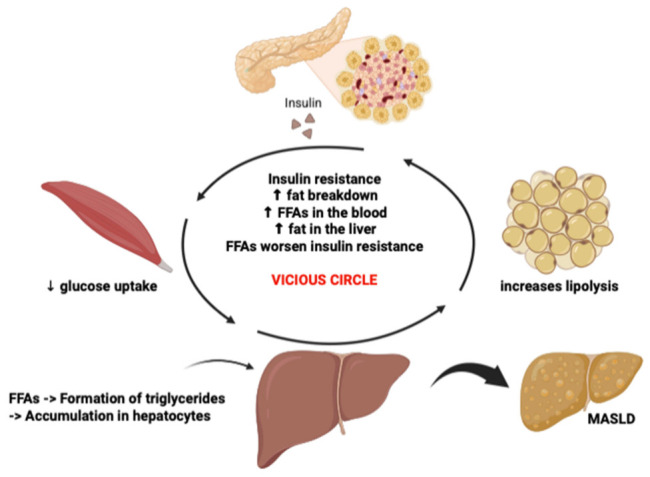
Pathophysiological link between insulin resistance and hepatic fat accumulation. Insulin resistance reduces glucose uptake in skeletal muscle and increases lipolysis in adipose tissue, leading to elevated circulating free fatty acids (FFAs). Excess FFAs are taken up by the liver, where they are re-esterified into triglycerides and accumulate in hepatocytes, promoting hepatic steatosis. Elevated FFAs further impair insulin signaling, creating a self-perpetuating vicious cycle that contributes to the development of MASLD.

In recent years, there has been a significant shift in the nomenclature of lipid accumulation-related liver disease, transitioning from non-alcoholic fatty liver disease (NAFLD) to metabolic dysfunction-associated steatotic liver disease (MASLD). This change reflects a deeper understanding of the disease’s pathophysiology, which is now recognized as being closely linked to components of metabolic syndrome, including type 2 diabetes mellitus (T2DM), visceral obesity, arterial hypertension, and dyslipidemia [1]. These metabolic disorders share a common pathogenic mechanism strongly associated with insulin resistance [2]. In both T2DM and MASLD, insulin resistance leads to increased hepatic glucose production and reduced glucose uptake in skeletal muscle. Additionally, it promotes decreased inhibition of lipolysis in adipose tissue and enhanced hepatic de novo lipogenesis, resulting in elevated levels of circulating glucose and free fatty acids (FFAs) [3]. The surplus of circulating FFAs contributes to an increased influx of lipids into the liver, where they are primarily stored as triglycerides [2]. This hepatic lipid accumulation not only underlies steatosis but also induces cellular stress and metabolic derangement. Elevated plasma levels of FFAs further exacerbate insulin resistance by interfering with insulin signaling at the post-receptor level, thereby amplifying metabolic derangement. This creates a self-perpetuating vicious cycle in which insulin resistance promotes FFA accumulation, and FFA excess, in turn, exacerbates insulin resistance (Figure 1) [4]. To date, weight loss remains the only intervention consistently shown to improve histological features of MASLD, with a reduction of at least 7–10% in body weight associated with a significant decrease in hepatic fat content, inflammation, fibrosis, and hepatocellular injury [5,6]. Despite the recent FDA approval of resmetirom, a selective thyroid hormone receptor-β (THR-β) agonist, for the treatment of patients with metabolic dysfunction-associated steatohepatitis (MASH) and stage F2–F3 fibrosis, several practical considerations may limit its widespread clinical adoption. Resmetirom represents the first-in-class pharmacological therapy approved for MASH with fibrosis and, unlike many investigational agents, its mechanism of action is not primarily mediated through weight reduction. However, further post-marketing studies are warranted to better characterize its long-term efficacy and safety, particularly in patients with complex metabolic comorbidities [7]. More recently, attention has increasingly focused on the therapeutic role of incretin-based therapies in patients with metabolic disorders. Glucagon-like peptide-1 (GLP-1) is an incretin hormone secreted by intestinal enteroendocrine cells that modulates insulin and glucagon secretion, regulates food intake, and slows gastrointestinal motility, thereby contributing to postprandial glucose homeostasis [8]. GLP-1 receptor agonists (GLP-1 RAs) enhance glucose-dependent insulin secretion, suppress inappropriate glucagon release, delay gastric emptying, and reduce appetite and caloric intake, and are therefore strongly recommended for the management of T2DM [9]. Importantly, beyond their systemic metabolic effects, incretin-based therapies may exert direct hepatic actions. GLP-1 receptors have been identified not only in pancreatic β-cells but also in hepatocytes and other liver-resident cells, suggesting a potential role in the regulation of hepatic lipid metabolism. Activation of these receptors has been associated with reduced hepatic triglyceride accumulation, improved insulin signaling, and modulation of inflammatory pathways. In addition, incretin signaling may indirectly influence hepatic stellate cell activation and fibrogenesis through correction in metabolic homeostasis and reductions in lipotoxicity. In parallel, glucose-dependent insulinotropic polypeptide (GIP) receptors and glucagon receptors are increasingly recognized as relevant targets in the liver and adipose tissue, further supporting the development of multi-receptor agonists. Dual agonists of GIP and GLP-1 receptors (“twincretins”) and triple agonists targeting GIP, GLP-1, and glucagon (GCG) receptors represent a new therapeutic strategy, combining complementary metabolic and potentially direct hepatic effects [10,11,12]. Given that metabolic impairment underlies hepatic steatosis and its progression, this review critically examines the current clinical evidence on the efficacy of incretin-based therapies in patients with MASLD. Special attention is given to changes in biochemical markers (e.g., liver enzymes and inflammatory markers) and imaging-based assessments of hepatic steatosis and fibrosis, in order to better define the potential impact of these agents on the hepatic manifestations of metabolic disease. Importantly, because incretin-based therapies simultaneously target both systemic metabolic pathways and, potentially, intrahepatic processes, a clearer interpretation of their hepatic effects requires distinguishing between indirect effects mediated by weight loss and metabolic regulation and direct hepatic mechanisms. These domains are closely interconnected, yet their relative contribution appears to vary across different drug classes and remains incompletely defined, representing a key challenge in the interpretation of current evidence. For this reason, the interpretation of efficacy across incretin-based therapies requires a clear hierarchy of outcomes. Reductions in aminotransferase levels, improvements in non-invasive fibrosis scores, and decreases in liver fat content assessed by magnetic resonance imaging–proton density fat fraction (MRI-PDFF) should be regarded as biologically meaningful but surrogate signals of hepatic benefit. By contrast, paired-biopsy endpoints, including MASH resolution without worsening of fibrosis and without worsening of steatohepatitis, provide a higher level of evidence for disease modification. Finally, the ability of these agents to prevent cirrhosis, hepatic decompensation, hepatocellular carcinoma, liver transplantation, or liver-related mortality remains largely unproven and requires long-term outcome studies. This distinction is essential when comparing selective GLP-1 receptor agonists, dual incretin agonists, and triple agonists, as the apparent magnitude of anti-steatotic activity does not necessarily correspond to equivalent histological or clinical benefit.

A structured literature search was performed in PubMed, Embase, and other major databases using predefined keywords, including “MASLD”, “NAFLD”, “MASH”, “GLP-1 receptor agonists”, “GIP”, “dual agonists”, “triple agonists”, and “incretin-based therapies”. Studies were selected based on their relevance to clinical and metabolic outcomes, with priority given to randomized controlled trials, meta-analyses, and high-quality observational studies.

## 2. GLP-1 Receptor Agonists: An Emerging Treatment of Metabolic Syndrome and Its Complications

GLP-1 is a 30-amino acid incretin peptide derived from proglucagon, biologically active in its GLP-1 (7–36) amide form, with a molecular weight of approximately 3.3 kDa. It is rapidly degraded by dipeptidyl peptidase-4 (DPP-4), resulting in a short plasma half-life of approximately 1–2 min. Structurally, GLP-1 adopts an α-helical conformation and is C-terminally amidated, which enhances its metabolic stability. Pharmacologic GLP-1 receptor agonists incorporate chemical modifications, such as acylation or amino acid substitutions, to enhance resistance to enzymatic degradation and prolong systemic exposure. GLP-1 is synthesized by enteroendocrine L cells, predominantly in the distal ileum and colon, with lesser expression in the jejunum and duodenum. In the pancreas, GLP-1 enhances glucose-dependent insulin secretion from β-cells and suppresses glucagon secretion from α-cells [13]. Within the central nervous system, GLP-1 acts on the hypothalamus and brainstem to reduce appetite and promote weight loss. Although human data are limited, emerging evidence suggests reduced GLP-1 receptor expression in the hypothalamus of individuals with type 2 diabetes compared to non-diabetic controls. Endogenously produced GLP-1 in the central nervous system is believed to originate primarily from the brainstem and to be transported via axonal projections to the hypothalamus [14]. Experimental studies in humans demonstrate that GLP-1 enhances cerebral glucose uptake and reduces anticipatory food reward [15,16], both of which are associated with decreased food intake. Long-acting GLP-1 receptor agonists also delay gastric emptying and reduce gastric motility, although these effects tend to attenuate over time and are thought to play a secondary role in long-term weight and glycemic control [17]. Although not highly specific [18], hepatic cytolysis markers, particularly aspartate aminotransferase (AST) and, more prominently, alanine aminotransferase (ALT) serve as indirect indicators of possible hepatic inflammation in patients with MASLD. Elevated levels of these enzymes are associated with an increased risk of progression to MASH and eventually to cirrhosis [19]. Therapeutic interventions that slow progression have consistently been linked to significant reductions in hepatic cytolysis markers [19]. A growing body of evidence supports the beneficial role of glucagon-like peptide-1 receptor agonists (GLP-1 RAs) in reducing liver enzymes associated with hepatocellular injury [20].

Currently, weight reduction remains the only validated therapeutic approach for MASLD, typically obtained through lifestyle interventions (dietary modification and physical activity) or bariatric surgery. A sustained weight loss of ≥7–10% has been consistently associated with improvements in hepatic steatosis, inflammation, and fibrosis, and with reduced disease progression [5]. In addition to lifestyle modifications, treatment with GLP-1 RAs has been shown to induce a reduction in body weight ranging from 2 to 7 kg, primarily through the promotion of early satiety and appetite suppression, which ultimately decreases caloric intake [21,22]. Furthermore, these agents reduce food cravings and enhance the hedonic response to eating [23]. The mechanisms underlying these effects are both central and peripheral. Within the central nervous system, serotonin plays a pivotal role in appetite regulation. GLP-1 stimulation has been shown to reduce hypothalamic 5-HT2A receptor expression, thereby attenuating hunger perception [24]. Peripherally, GLP-1 RAs delay gastric emptying and slow intestinal motility, which reinforces the sensation of early satiety transmitted to the brain [25]. However, prolonged receptor activation leads to tachyphylaxis, progressively diminishing the effect on gastric emptying over time [26]. The magnitude of weight loss achieved is highly dependent on the specific GLP-1 RA and the administered dose. While glycemic control is often attained with relatively low doses, more substantial reductions in body weight typically require higher drug doses [27]. Notably, interindividual variability in weight-loss response among patients receiving GLP-1 RAs is significantly greater than that observed in glycemic outcomes [28]. Liraglutide is a GLP-1 analog sharing 97% structural homology with human GLP-1, an incretin hormone secreted by the gut. Unlike native GLP-1, which has a very short elimination half-life of 1–2 min, liraglutide has a markedly prolonged half-life of approximately 13 h, allowing once-daily subcutaneous administration [29,30]. Originally developed for the treatment of type 2 diabetes mellitus, liraglutide has consistently demonstrated improvements in glycemic control at doses up to 1.8 mg/day [31,32]. In addition to lowering glycated hemoglobin (HbA1c) concentrations [32,33], liraglutide has been shown to enhance pancreatic β-cell function [34] and reduce systolic blood pressure [32]. Importantly, because it induces dose-dependent weight loss, liraglutide represents an attractive therapeutic option not only for type 2 diabetes but also for obesity. The effects of liraglutide on body weight are supported by both clinical and preclinical studies. Experimental evidence in minipigs and rats has demonstrated reductions in food intake and subsequent weight loss with liraglutide administration [35]. In the minipig obesity model, treatment resulted in a measurable decrease in feeding frequency and meal size [35]. These observations align with the distribution of GLP-1 receptors within several brainstem nuclei that are critically involved in appetite regulation [36], suggesting that subcutaneously administered liraglutide may act directly on central pathways governing hunger and satiety. Based on these findings, the primary aim of clinical investigations has been to evaluate the impact of liraglutide, at doses up to 3.0 mg per day, on body weight reduction in obese individuals when combined with an energy-deficient low-fat diet and structured physical activity counseling. Indeed, several studies conducted over the past decade have demonstrated that liraglutide induces significant weight loss, lowers the incidence of prediabetes, and improves obesity-related risk factors, independently of the presence of type 2 diabetes mellitus [37,38,39,40,41]. After one year of treatment with a daily dose of 3 mg, a mean body weight reduction of 8.4 kg has been reported [22]. When comparing data across clinical trials [22,37,42,43], the average weight loss ranges between 3.4% and 6.1% of total body weight. Notably, among treated patients, approximately two-thirds achieve a weight reduction of at least 5%, while about one-third experience a weight loss greater than 10% [22]. The anorectic effect of liraglutide appears very early; in animal studies, a significant decrease in food intake was observed within 1 h of administration [44]. Similarly, another second-generation GLP-1 analog that has been extensively investigated is semaglutide. This agent was specifically engineered to overcome the rapid degradation of the endogenous peptide through targeted amino acid substitutions and the attachment of a C18 fatty acid chain, which strengthens albumin binding. These modifications extend their half-life to approximately seven days, thereby enabling once-weekly administration, with an oral formulation also available. In comparison with liraglutide, which requires daily dosing, semaglutide exhibits a more favorable pharmacokinetic profile and has consistently shown superior efficacy in improving glycemic control and achieving greater weight loss, positioning it as a major advancement in GLP-1 receptor agonist therapy [45]. Semaglutide is a second-generation GLP-1 receptor agonist available in two formulations: an oral preparation requiring daily administration and a subcutaneous formulation administered once weekly [46]. Multiple clinical studies have demonstrated that semaglutide reduces energy intake and achieves greater weight loss than placebo, both in adults [21,47,48,49] and adolescents [50]. At a weekly dose of 2.4 mg, approved for the treatment of obesity, semaglutide has been shown to induce an average weight reduction of 9.6% to 17.4%, accompanied by decreases in waist circumference and blood pressure, and improvements in lipid profile [48,51]. Compared with other GLP-1 receptor agonists, semaglutide exerts a more pronounced weight-lowering effect [52]. At doses equal to or exceeding 0.2 mg/day (≈1.4 mg/week), semaglutide has demonstrated greater efficacy than liraglutide, irrespective of the liraglutide dose [21]. Particularly compelling evidence comes from the STEP 8 randomized trial [45], in which semaglutide 2.4 mg/week produced a mean weight loss of 15.8% after 68 weeks of treatment, compared with 6.4% observed with liraglutide. Moreover, 70.9% of patients treated with semaglutide achieved weight loss greater than 10%, a therapeutic target in NAFLD, versus 25.6% of those receiving liraglutide, with comparable rates of gastrointestinal adverse events in both groups [45]. Based on this evidence, both liraglutide and semaglutide have been approved for the treatment of obesity in addition to type 2 diabetes mellitus, and their use is indicated in both diabetic and non-diabetic individuals [28,53]. For obesity, the maximum approved dose of liraglutide is 3 mg/day (versus 1.8 mg/day for diabetes), whereas semaglutide is approved up to 2.8 mg/week (≈0.4 mg/day) for obesity and 1 mg/week for T2DM. The weight-lowering effect of these agents, together with their ability to reduce systemic inflammation in obese individuals and improvements in glycemic and metabolic profiles, suggests that this drug class may provide additional benefits in patients with MASLD. Given that MASLD is, by definition, a dysmetabolic condition, close monitoring of the metabolic component is essential, as its correction is the primary therapeutic target of the disease. However, it is important to note that these agents are not devoid of adverse effects. In non-diabetic patients with overweight or obesity, GLP-1 receptor agonists have been associated with an increased incidence of gastrointestinal adverse events, particularly nausea, vomiting, diarrhea, and constipation. Therefore, careful consideration of these risk profiles is essential to inform therapeutic decisions and ensure an appropriate balance between potential benefits and risks [54].

## 3. Effect of GLP-1 RA on MASLD

In recent years, several lines of evidence have investigated the use of GLP-1 receptor agonists in patients with MASLD, aiming to assess their efficacy on steatosis, fibrosis, and hepatocellular injury, as measured by markers of hepatic cytolysis. From a mechanistic perspective, the hepatic effects of GLP-1 receptor agonists can be interpreted within a dual framework, in which these agents act predominantly through indirect mechanisms mediated by weight loss and systemic metabolic improvement, alongside potential direct hepatic mechanisms involved in lipid metabolism and inflammatory regulation. At the same time, scientific interest has progressively expanded to the gut–liver axis, which is now recognized as a key element in the pathophysiology of the disease. Consolidated evidence has demonstrated the presence of specific gut dysbiosis associated with MASLD and its different stages [55]. During the progression of liver disease, serum levels of aspartate aminotransferase (AST) and, more importantly, alanine aminotransferase (ALT) serve as markers of cytolysis and represent indicators of progression toward inflammatory disease, ultimately culminating in cirrhosis [19]. In overweight and obese patients with MASLD, achieving a weight loss of ≥7–10% is consistently associated with improvements in liver enzyme levels and with histological amelioration of the disease [56]. Moreover, a growing body of evidence strongly supports the beneficial role of GLP-1 receptor agonists in reducing hepatic cytolysis markers [49,57] (Table 1). In a 2013 prospective randomized study, Fan H. et al. assigned 117 patients with T2DM and NAFLD to receive either exenatide or metformin for 12 weeks. Both treatments improved metabolic and hepatic parameters, but exenatide achieved greater reductions in BMI, liver enzymes, and inflammatory markers, along with more pronounced increases in the AST/ALT ratio and adiponectin [58]. In addition, another study involved 19 patients with NASH who initially underwent a 24-week lifestyle modification program, after which treatment with liraglutide at a dose of 0.9 mg/day was initiated for an additional 24 weeks. This approach resulted in significant improvements in body mass index, visceral fat accumulation, aminotransferases, and glucose abnormalities. In a subgroup of 10 patients who continued liraglutide therapy for up to 96 weeks and underwent repeat liver biopsy, six showed a reduction in histological inflammation, documented by improvements in the NASH activity score and in fibrosis stage according to the Brunt classification [59]. Another study compared the effects of gliclazide, liraglutide, and metformin in patients with T2DM and NAFLD. In this randomized controlled trial, 87 subjects were assigned to receive liraglutide, metformin, or gliclazide for 24 weeks. Primary outcomes included HbA1c levels, intrahepatic fat (IHF) content, and liver function. The results showed that, in T2DM patients with NAFLD, gliclazide produced less pronounced improvements in liver function, IHF reduction, HbA1c levels, and weight loss compared with liraglutide and metformin. Moreover, liraglutide provided slightly greater benefits than metformin [60]. In addition, a retrospective study by Seko et al. evaluated the efficacy of dulaglutide in patients with biopsy-proven NAFLD and type 2 diabetes mellitus refractory to dietary intervention. Fifteen patients were treated with dulaglutide 0.75 mg once weekly for 12 weeks; two subjects were excluded from the final analysis. After treatment, not only body weight and hemoglobin A1c (HbA1c), but also transaminase levels were significantly reduced. Furthermore, total body fat mass and liver stiffness decreased, supporting the potential benefits of dulaglutide on both metabolic and hepatic parameters [61]. Another important study conducted by Cusi et al. evaluated the effects of dulaglutide compared with placebo on liver function as well as glycemic and metabolic parameters in patients with type 2 diabetes, including a subgroup with NAFLD or NASH. This analysis included a total of 1499 participants pooled from the AWARD-1, AWARD-5, AWARD-8, and AWARD-9 clinical trials (dulaglutide 1.5 mg, *n* = 971; placebo, *n* = 528). After 6 months of treatment, dulaglutide was associated with a significant reduction in alanine aminotransferase (ALT), aspartate aminotransferase (AST), and gamma-glutamyl transpeptidase (γ-GT) levels compared with placebo, indicating a beneficial effect on liver function [62]. Another prospective, double-blind study evaluated the efficacy of semaglutide in a 72-week, phase 2 trial conducted in patients with biopsy-confirmed NASH and liver fibrosis at stages F1, F2, or F3. A total of 320 patients (230 with stage F2 or F3 fibrosis) were randomized to receive semaglutide at doses of 0.1 mg (80 patients), 0.2 mg (78 patients), or 0.4 mg (82 patients) or placebo (80 patients). NASH resolution without worsening fibrosis was achieved in 40% of patients treated with 0.1 mg of semaglutide, 36% with 0.2 mg, and 59% with 0.4 mg, compared with 17% in the placebo group (*p* < 0.001 for semaglutide 0.4 mg vs. placebo). This phase 2 study, therefore, demonstrated that treatment with semaglutide resulted in a significantly higher rate of NASH resolution compared with placebo [49]. In conclusion, it is important to emphasize that most of the available evidence relies on surrogate endpoints such as reductions in transaminase levels, improvements in metabolic parameters, or changes observed through imaging rather than on robust and systematically assessed histological outcomes. Within this context, the observed hepatic benefits are likely driven predominantly by indirect metabolic effects related to weight loss, while the contribution of direct hepatic mechanisms remains less clearly defined. This methodological limitation inevitably weakens the strength and accuracy of these findings, highlighting the need for further prospective studies with histological confirmation to more definitively establish the true impact of GLP-1 receptor agonists on liver disease progression.

## 4. Effect of Dual GLP-1 + GIP Agonist on MASLD

Tirzepatide (LY3298176) is a synthetic 39–amino acid linear peptide engineered to act as a dual agonist of the glucose-dependent insulinotropic polypeptide receptor (GIPR) and the glucagon-like peptide-1 receptor (GLP-1R). The molecule contains two non-standard amino acids (α-aminoisobutyric acid, Aib) at positions 2 and 13 and is modified with a C20 fatty di-acid moiety attached to lysine at position 20 through a linker, enabling reversible binding to albumin and allowing once-weekly subcutaneous administration [63]. Tirzepatide was designed by incorporating GLP-1-like activity into the GIP peptide backbone, resulting in a dual incretin receptor agonist with a signaling bias toward GIPR activation. The molecule binds the GIP receptor with an affinity comparable to native GIP, whereas its affinity for the GLP-1 receptor is approximately five-fold lower than that of native GLP-1. This pharmacological profile allows effective activation of GIPR while potentially minimizing GLP-1-related gastrointestinal adverse effects [64]. At the receptor level, tirzepatide differentially modulates GIPR and GLP-1R signaling and internalization. While it acts as a full agonist for GIPR internalization, inducing receptor internalization to an extent similar to native GIP, it shows markedly reduced efficacy in promoting GLP-1R internalization, reaching less than 40% of the maximal effect observed with GLP-1. This reduced activity is consistent with weaker β-arrestin recruitment. In addition, kinetic studies demonstrate that tirzepatide elicits a monophasic cAMP response at GLP-1R, whereas native GLP-1 produces a biphasic signaling profile, suggesting differences in the temporal regulation of receptor signaling. These distinctive pharmacodynamic properties may contribute to the unique metabolic effects of tirzepatide [64]. Preclinical studies have demonstrated that tirzepatide exerts beneficial effects on MASLD, a condition characterized by dysregulated lipid metabolism, insulin resistance, inflammation, and oxidative stress. In mouse models of MASLD induced by a high-fat diet (HFD) or a high-fat, high-fructose/cholesterol diet (HFFC), weekly administration of tirzepatide significantly attenuated body weight gain and reduced both body and liver weight. Histological analysis, including hematoxylin–eosin and Oil Red O staining, revealed substantial improvements in hepatic steatosis and hepatocellular injury. Similar anti-steatotic effects were observed in vitro, where tirzepatide reduced palmitic acid-induced lipid accumulation in HepG2 cells [65]. Mechanistically, tirzepatide reduces hepatic lipid accumulation through multiple pathways. The drug significantly lowers blood glucose levels and reduces hepatic triglyceride and cholesterol content. It also downregulates proteins involved in mitochondrial oxidative phosphorylation, including Cyc, COX4, and OPA1, suggesting modulation of mitochondrial metabolic activity. In addition, tirzepatide suppresses the expression of fatty acid uptake mediators, such as CD36 and OBP2A, thereby limiting lipid influx into hepatocytes. Notably, this regulation appears to be tissue-specific, as CD36 expression in skeletal muscle remains unaffected [65]. Further multi-omics analyses have demonstrated that tirzepatide modulates several metabolic pathways relevant to MASLD progression. In HFD-fed mice, tirzepatide downregulated fatty acid transport proteins, including CD36 and FABP2/4, enhanced mitochondrial-lysosomal function by upregulating LAMP1 and LAMP2, and promoted cholesterol efflux by regulating HNF4A, ABCG5, and ABCG8. These coordinated changes reshaped hepatic metabolomic and lipidomic profiles and contributed to improved liver function [66]. Additional evidence suggests that the gut–liver axis also contributes to tirzepatide’s liver-related metabolic effects. In a diabetic mouse model induced by a high-fat diet and streptozotocin, tirzepatide improved hepatic steatosis, insulin resistance, and lipid metabolism, while restoring intestinal barrier integrity and increasing the expression of tight junction proteins such as occludin and ZO-1. Importantly, tirzepatide reversed diet-induced gut microbiota dysbiosis, markedly increasing the abundance of *Akkermansia*, a bacterial genus associated with improved metabolic health. Depletion of gut microbiota with antibiotics substantially attenuated the hepatic benefits of tirzepatide, indicating a key role for the microbiome in mediating these effects [67]. Translating these mechanistic and preclinical observations into clinical settings, the SURPASS-3 MRI substudy investigated the effects of tirzepatide on liver fat content and abdominal adipose tissue distribution in patients with type 2 diabetes. This randomized phase 3 trial compared once-weekly tirzepatide (5, 10, or 15 mg) with insulin degludec over 52 weeks, using magnetic resonance imaging–proton density fat fraction (MRI-PDFF) to quantify liver fat content and assess changes in visceral and subcutaneous adipose tissue [68]. The study demonstrated that tirzepatide significantly reduced liver fat content compared with insulin degludec. Participants treated with tirzepatide 10 mg and 15 mg showed a mean absolute improvement in hepatic steatosis of approximately −8.1% from baseline, compared with −3.4% in the insulin degludec group. In addition, a substantially higher proportion of patients receiving tirzepatide achieved a ≥30% decrease in liver fat, a threshold associated with meaningful histological improvement in steatohepatitis. Tirzepatide treatment was also associated with significant reductions in visceral adipose tissue and abdominal subcutaneous adipose tissue, indicating broader improvements in body fat distribution and metabolic status [68]. Further clinical evidence supporting the hepatoprotective potential of tirzepatide comes from the phase 2 SYNERGY-NASH trial, which evaluated tirzepatide in patients with MASH and moderate-to-severe liver fibrosis. Over a 52-week treatment period, tirzepatide significantly increased the proportion of patients achieving resolution of steatohepatitis without worsening of fibrosis compared with placebo. These histological improvements were accompanied by substantial weight loss and improved glycemic control, highlighting the systemic metabolic benefits of incretin-based therapies in patients with MASH [69]. Additional clinical observations have also demonstrated improvements in liver-related parameters following tirzepatide treatment, including a decrease in hepatic fat accumulation, aminotransferase levels, and surrogate markers of fibrosis, further supporting its potential role as a candidate therapeutic strategy for MASLD and related metabolic liver diseases [70]. However, these findings should be interpreted according to the level of evidence provided by each endpoint. The SURPASS-3 MRI substudy offers strong metabolic and imaging-based evidence, but MRI-PDFF reduction remains a surrogate marker of decreased hepatic fat content and does not, by itself, establish MASH resolution or fibrosis regression [68]. In contrast, the SYNERGY-NASH trial provides a higher level of evidence because it evaluated biopsy-based histological endpoints, showing that tirzepatide increased MASH resolution without worsening of fibrosis compared with placebo [69]. Even so, these data derive from a phase 2 trial with a 52-week treatment period, and longer phase 3 studies are still needed to determine whether the observed histological improvements translate into fewer liver-related clinical events. Therefore, tirzepatide should be viewed as one of the most promising dual incretin agonists for MASH, but its clinical positioning remains less mature than that of agents supported by phase 3 histological and regulatory evidence. From a conceptual standpoint, the hepatic effects of tirzepatide can be interpreted within a dual framework. Indirect mechanisms appear to play a dominant role, with substantial weight loss, improvements in insulin resistance, and decrease in central fat mass, representing key drivers of decreased hepatic fat content and improved metabolic parameters. At the same time, preclinical and translational evidence suggests the presence of direct hepatic actions, including modulation of fatty acid transport, mitochondrial function, and lipid oxidation pathways. While these findings support a component of intrahepatic metabolic remodeling, their relative contribution in humans remains incompletely defined. Compared with selective GLP-1 receptor agonists, which appear to act predominantly through weight loss-mediated mechanisms, tirzepatide may exert a more integrated metabolic effect, combining systemic improvements with potential direct hepatic modulation. Beyond its metabolic and anti-steatotic effects, emerging evidence suggests that tirzepatide may also influence pathways involved in hepatic carcinogenesis. In a mouse model of MASLD that recapitulates the full spectrum of disease progression—from steatosis and steatohepatitis to advanced fibrosis and hepatocellular carcinoma (HCC)—treatment with tirzepatide significantly attenuated disease progression [71]. Pharmacological treatment with tirzepatide markedly reduced hepatic steatosis, inflammatory infiltration, and fibrotic deposition, and significantly decreased tumor burden. Notably, the timing of treatment appeared critical: early administration of tirzepatide, initiated before the development of advanced fibrosis and tumorigenesis, completely prevented hepatocellular carcinoma, whereas treatment initiated after fibrosis had already developed and still resulted in a substantial reduction in tumor burden [71]. At the molecular level, tirzepatide downregulated oncogenic signaling pathways, particularly Wnt/β-catenin signaling. In parallel, the drug modulated the expression of genes involved in cell proliferation, survival, and metabolic reprogramming, while restoring pathways related to lipid metabolism, mitochondrial function, and inflammatory responses. Collectively, these findings suggest that tirzepatide may limit the molecular drivers of hepatic carcinogenesis and that early correction of metabolic dysfunction could be critical in preventing progression from MASLD to HCC [71]. Table 2 summarizes studies of Dual GLP-1 + GIP agonist’s effect on MASLD.

## 5. Effect of Dual GLP-1 + Glucagon Agonist on MASLD

Glucagon, primarily secreted by pancreatic α-cells and, to a lesser extent, by intestinal mucosa, plays a central role in systemic energy homeostasis. Beyond its classical effects on glucose metabolism, namely stimulation of gluconeogenesis and glycogenolysis, glucagon exerts pleiotropic actions on hepatic lipid metabolism and mitochondrial function that are highly relevant in MASH. At the hepatic level, glucagon receptor activation enhances mitochondrial turnover and oxidative capacity, reduces oxidative stress, suppresses de novo lipogenesis, and decreases intrahepatic lipid accumulation. Additionally, glucagon reduces hepatic glycogen content and promotes metabolic flexibility, a feature particularly relevant in the context of chronic energy surplus that characterizes MASH. Importantly, glucagon signaling has been shown to attenuate hepatic stellate cell activation, suggesting a potential antifibrotic effect [72]. The rationale for dual GLP-1R/GCGR agonism lies in the complementary metabolic effects of these two hormonal pathways. GLP-1 receptor activation enhances glucose-dependent insulin secretion, suppresses appetite, and promotes weight loss, thereby improving systemic metabolic derangement. Conversely, glucagon receptor activation stimulates hepatic fatty acid oxidation, increases energy expenditure, and reduces de novo lipogenesis in hepatocytes. The combined activation of these receptors allows simultaneous improvement in glycemic control, weight control, and hepatic lipid metabolism, potentially overcoming some limitations of GLP-1 receptor agonism alone [73]. This dual agonism reflects the integration of two complementary mechanisms of action. Indirect effects mediated by weight loss and improved metabolic control remain important; however, compared with selective GLP-1 receptor agonists, this class provides a more prominent contribution of direct hepatic mechanisms. In particular, glucagon receptor activation directly enhances hepatic fatty acid oxidation, increases energy expenditure, and suppresses de novo lipogenesis, thereby targeting intrahepatic lipid accumulation more directly. These mechanistic considerations have provided the foundation for the development of pharmacological agents that simultaneously activate GLP-1 and glucagon receptors. Accordingly, several GLP-1/glucagon receptor dual agonists have recently entered clinical development, and emerging clinical evidence suggests that these compounds may exert beneficial effects on hepatic steatosis, metabolic dysfunction, and potentially disease progression in MASLD and MASH. Cotadutide (MEDI0382) is among the most extensively studied GLP-1/glucagon receptor co-agonists. It has been engineered to favor GLP-1R activity while maintaining sufficient glucagon receptor activation to promote hepatic lipid oxidation [74]. In a randomized phase 2b clinical trial involving patients with overweight or obesity and type 2 diabetes, cotadutide demonstrated significant improvements in glycemic control and body weight over 54 weeks. Importantly, post hoc analyses showed favorable changes in hepatic biomarkers and indices of steatosis, supporting its potential role in the treatment of steatohepatitis [75]. Further evidence suggests that cotadutide may exert direct hepatic benefits beyond weight reduction. Studies comparing cotadutide with liraglutide have shown greater decrease in hepatic steatosis despite similar degrees of weight loss, suggesting additional liver-specific metabolic effects mediated by glucagon receptor signaling [76]. Efinopegdutide (MK-6024) is another investigational GLP-1/glucagon receptor dual agonist currently under clinical development for MASLD and MASH. In a phase 2a randomized study in patients with NAFLD, efinopegdutide significantly reduced liver fat content measured by MRI-PDFF. Notably, treatment with efinopegdutide produced an improvement of hepatic fat deposition compared with semaglutide, a selective GLP-1 receptor agonist, highlighting the potential added benefit of glucagon receptor activation [77]. The observed reduction in hepatic steatosis likely reflects both indirect effects mediated by weight loss and direct hepatic mechanisms such as enhanced fatty acid oxidation and decreased lipogenesis [77]. Ongoing clinical trials are evaluating the ability of efinopegdutide to induce histological resolution of MASH without progression of fibrosis [78]. More recently, additional dual agonists have entered clinical development. Pemvidutide, a long-acting GLP-1/glucagon receptor co-agonist, has demonstrated clinically relevant results in patients with MASLD. In a phase 2 trial, weekly administration of pemvidutide resulted in significant reductions in liver fat content, body weight, and biomarkers of hepatic inflammation compared to placebo [79].

Taken together, GLP-1/glucagon receptor co-agonists provide a biologically attractive model because glucagon receptor activation may more directly influence hepatic lipid oxidation, energy expenditure, and de novo lipogenesis than selective GLP-1 receptor agonism. Nevertheless, the current clinical evidence for this class remains largely based on reductions in liver fat measured by MRI-PDFF, improvements in aminotransferases, and changes in non-invasive indices. These endpoints are useful for detecting anti-steatotic and metabolic activity, but they should not be interpreted as definitive evidence of fibrosis regression or long-term disease modification. Therefore, compared with semaglutide and tirzepatide, GLP-1/glucagon receptor co-agonists should currently be regarded as mechanistically compelling but still clinically less mature for MASH-specific treatment. Table 3 summarizes studies of Dual GLP 1 + glucagon agonist’s effect on MASLD.

## 6. Triple Agonists Targeting GLP-1, GIP, and Glucagon Receptors

More recently, triple agonists targeting GLP-1, GIP, and glucagon receptors have emerged as a potential new therapeutic approach, aiming to maximize metabolic efficacy through complementary mechanisms of action [80]. The rationale behind triple agonism lies in the complementary metabolic roles of the three targeted receptors. GLP-1 receptor activation promotes glucose-dependent insulin secretion, delays gastric emptying, and reduces appetite through central satiety pathways, ultimately improving glycemic control and inducing weight loss. GIP receptor agonism enhances insulin secretion and may improve adipose tissue metabolism and insulin sensitivity. Meanwhile, activation of the glucagon receptor increases hepatic energy expenditure, stimulates lipolysis, and promotes thermogenesis [81]. Although glucagon historically raised concerns due to its hyperglycemic effects, its combination with GLP-1 receptor agonism appears to mitigate this drawback. GLP-1-mediated insulinotropic effects counterbalance glucagon-induced increases in hepatic glucose production, while glucagon receptor stimulation enhances energy expenditure and lipid oxidation. This synergistic metabolic interaction represents the theoretical advantage of triple agonists compared with mono- or dual-agonist therapies [80]. In this context, the effects of triple agonists can be interpreted as the result of a highly integrated interaction between indirect and direct mechanisms. Profound weight loss and improvements in insulin resistance represent major drivers of metabolic benefit; however, concomitant activation of the glucagon receptor also supports direct hepatic effects, particularly through enhanced lipid oxidation and increased energy expenditure. Compared with dual agonists, this class may therefore provide a more comprehensive metabolic reprogramming, although the relative contribution of individual pathways remains difficult to disentangle in clinical settings. The most advanced compound within this therapeutic class is retatrutide, a once-weekly injectable peptide that simultaneously activates GLP-1, GIP, and glucagon receptors. Early clinical trials have demonstrated remarkable metabolic effects. In a phase 2 randomized clinical trial involving patients with obesity, retatrutide produced dose-dependent weight loss reaching up to 24.2% at 48 weeks, substantially exceeding the placebo response. Notably, between 36% and 48% of patients receiving the highest doses achieved at least 25% weight reduction [82]. In addition to weight loss, retatrutide significantly improved multiple cardiometabolic parameters, including fasting plasma glucose, HbA1c, waist circumference, and blood pressure [83]. Given the close relationship between obesity, insulin resistance, and hepatic steatosis, triple incretin agonists have also attracted considerable interest in hepatology. In a phase 2a randomized trial conducted by Sanyal et al. (2024) [84], retatrutide demonstrated marked effects on hepatic steatosis in patients with MASLD. Treatment led to substantial, dose-dependent decrease in liver fat content, as measured by MRI-PDFF, with relative decreases of approximately 80–86% after 48 weeks. At the highest doses, a large proportion of participants achieved normalization of hepatic fat content (<5%), indicating near-resolution of steatosis in many treated patients [84]. These improvements in hepatic fat were accompanied by significant reductions in serum aminotransferases levels, suggesting an improvement of liver inflammation. The hepatic benefits observed with retatrutide likely result from a combination of mechanisms, including pronounced weight loss, improved insulin sensitivity, and enhanced hepatic lipid oxidation related to glucagon receptor activation [85]. Collectively, these findings indicate that triple incretin agonism may exert powerful effects on hepatic steatosis and support the potential role of retatrutide as a therapeutic strategy for MASLD, although larger, longer trials with histological endpoints are still required to determine its impact on steatohepatitis and fibrosis. This distinction is particularly important for retatrutide. The marked reduction in hepatic fat observed with MRI-PDFF suggests a potent anti-steatotic effect and supports further development in MASLD; however, normalization of liver fat content cannot be assumed to represent histological MASH resolution, fibrosis regression, or prevention of liver-related outcomes. At present, the evidence for triple agonists should therefore be considered highly promising but primarily imaging-based, and their place in the therapeutic algorithm will depend on future biopsy-based and long-term clinical outcome trials [84]. The safety profile of triple agonists appears broadly consistent with that observed with other incretin-based therapies. The most common adverse events are gastrointestinal, including nausea, vomiting, and diarrhea, particularly during the dose-escalation phase [86]. Table 4 summarizes studies of triple agonist’s effect on MASLD.

### Comparative Synthesis of Incretin-Based Therapeutic Classes

When the available evidence is considered across therapeutic classes, several differences emerge in terms of mechanism, endpoint maturity, and clinical interpretability. Selective GLP-1 receptor agonists represent the most clinically established class, with benefits largely driven by weight loss, improved insulin sensitivity, reduced visceral adiposity, and favorable cardiometabolic effects. Within this class, semaglutide has generated the strongest histological evidence, progressing from phase 2 biopsy-based data showing NASH resolution without worsening of fibrosis to phase 3 evidence in patients with non-cirrhotic MASH and F2–F3 fibrosis [49,50]. Therefore, GLP-1 receptor agonists currently provide the clearest bridge between metabolic risk modification and liver-directed therapeutic benefit.

Dual GIP/GLP-1 receptor agonism appears to amplify the metabolic component of incretin therapy. Tirzepatide produces substantial weight loss and marked improvements in glycemic control, visceral adiposity, and liver fat content. Importantly, the SYNERGY-NASH trial moved this class beyond purely surrogate endpoints by demonstrating biopsy-based MASH resolution without worsening of fibrosis [69]. However, compared with semaglutide, the evidence remains at an earlier stage of clinical development for MASH-specific treatment, and longer phase 3 trials are required before definitive conclusions can be drawn regarding fibrosis regression and clinical outcomes. By contrast, GLP-1/glucagon receptor co-agonists and triple GLP-1/GIP/glucagon receptor agonists may offer a stronger mechanistic rationale for direct hepatic metabolic modulation, particularly through glucagon-mediated enhancement of lipid oxidation and energy expenditure. Agents such as cotadutide, efinopegdutide, pemvidutide, and retatrutide have shown substantial reductions in liver fat content and improvements in liver enzymes or metabolic biomarkers. Nevertheless, much of this evidence remains based on MRI-PDFF or biochemical endpoints. These findings support anti-steatotic activity but do not yet establish histological MASH resolution, fibrosis regression, or prevention of clinically meaningful liver-related events. Thus, the apparent potency of liver fat reduction should not be equated with the strength of evidence for disease modification. From a comparative perspective, semaglutide currently provides the most mature GLP-1 receptor agonist evidence for MASH, tirzepatide represents the most advanced dual incretin agonist with biopsy-based phase 2 efficacy, and glucagon-containing dual or triple agonists remain promising but less clinically validated. This hierarchy supports a cautious interpretation of efficacy and reinforces the need to distinguish metabolic improvement, anti-steatotic effects, histological response, and long-term clinical benefit.

## 7. Limitations of Current Evidence

Despite the encouraging results reported so far, the current body of evidence regarding the use of incretin-based therapies in MASLD still presents several important limitations. Many studies evaluating the hepatic effects of these agents rely on surrogate endpoints, such as reductions in aminotransferase levels or liver fat content assessed by imaging techniques, rather than on histological outcomes obtained through liver biopsy, which remains the gold standard for evaluating disease activity and fibrosis progression [87]. In addition, the use of these therapies in patients with advanced liver disease raises potential concerns, as hepatic dysfunction may alter drug pharmacokinetics and only limited safety data are currently available for this population. Another relevant issue is the substantial heterogeneity across available studies in terms of design, patient populations, and treatment duration, which complicates the interpretation and comparability of findings and limits the generalizability of results to the broader MASLD population [88]. Moreover, most clinical trials have relatively short follow-up periods, making it difficult to determine whether the observed improvements in hepatic steatosis and metabolic parameters translate into long-term clinical benefits, such as fibrosis regression or the prevention of cirrhosis, hepatocellular carcinoma, and liver-related mortality. Finally, although incretin-based therapies are generally well tolerated, they are not devoid of adverse effects. Gastrointestinal symptoms, including nausea, vomiting, and diarrhea, represent the most commonly reported events and may influence treatment adherence in some patients [89]. Taken together, these considerations highlight the need for large-scale randomized controlled trials with longer follow-up and histological endpoints to better define the long-term efficacy and safety of incretin-based therapies in the management of MASLD.

## 8. Direct Antifibrotic Mechanisms of Incretin-Based Therapies: Beyond Weight Loss

Although weight reduction remains a major determinant of histological improvement in MASLD/MASH, accumulating evidence suggests that incretin-based therapies may also exert antifibrotic effects through mechanisms that extend beyond body-weight loss alone. Liver fibrosis in MASLD is driven by a complex interplay among steatotic hepatocytes, inflammatory macrophages, and activated hepatic stellate cells (HSCs), and therefore drugs capable of interrupting this fibro-inflammatory crosstalk may attenuate disease progression independently of their anorectic action [90]. In this context, GLP-1 receptor agonists appear to reduce the upstream drivers of fibrogenesis by improving insulin resistance, decreasing lipotoxic hepatocellular stress, limiting oxidative damage, and dampening inflammatory signaling pathways that promote HSC activation and extracellular matrix deposition [90]. Experimental studies have shown that liraglutide can suppress NLRP3 inflammasome activation and hepatocyte pyroptosis through restoration of mitophagy, while also promoting Kupffer cell polarization toward an anti-inflammatory M2 phenotype via cAMP–PKA–STAT3 signaling [91,92]. These effects may reduce the release of profibrogenic mediators such as TNF-α, IL-1β, and TGF-β, thereby weakening the paracrine signals that sustain stellate cell activation. Additional preclinical data suggest that GLP-1 receptor agonists may directly interfere with HSC activation by inhibiting p38 MAPK-dependent signaling and collagen synthesis [93]. However, this issue remains controversial, since recent work in primary human hepatocytes and HSCs failed to demonstrate clear direct GLP-1- or GIP-mediated cellular responses, implying that a substantial proportion of the hepatic benefit may arise indirectly through systemic and intrahepatic metabolic remodeling rather than through a classical receptor-mediated effect in all hepatic cell populations [94]. This broader antifibrotic concept is further supported by emerging evidence on next-generation incretin co-agonists. In preclinical models, the GLP-1/glucagon dual agonist cotadutide promoted resolution of steatohepatitis and hepatic fibrosis through coordinated effects on mitochondrial function, hepatic lipogenesis, and energy metabolism, suggesting that restoration of hepatocellular metabolic fitness may secondarily deactivate fibrogenic pathways [95]. In parallel, semaglutide has recently been shown to reduce hepatic expression of fibrosis- and inflammation-related gene programs in experimental MASH models, supporting the hypothesis that incretin signaling can remodel the molecular architecture of fibroinflammation even before advanced fibrosis regresses histologically [96]. Clinically, semaglutide increased NASH resolution without worsening fibrosis, whereas tirzepatide significantly improved MASH resolution without fibrosis worsening in patients with moderate-to-severe fibrosis, findings that are consistent with an antifibrotic trajectory likely mediated by a combination of profound metabolic correction and attenuation of hepatic inflammatory injury [97,98]. Nonetheless, whether incretin-based therapies can consistently induce true fibrosis regression through direct cellular antifibrotic mechanisms in humans remains to be fully established, and this question should be addressed in future trials integrating paired liver histology with transcriptomic and proteomic profiling [99].

## 9. Future Perspectives

Future strategies in the management of MASLD should increasingly move toward a phenotype-driven and precision medicine-based approach, overcoming the traditional “one-size-fits-all” paradigm. One of the most relevant distinctions emerging in the recent literature is that between obese/overweight MASLD and normal-weight (lean) MASLD, as these phenotypes appear to differ not only in anthropometric characteristics but also in metabolic drivers, pathophysiological mechanisms, and clinical outcomes. Growing evidence suggests that lean MASLD represents a distinct clinical entity, with long-term outcomes that may be comparable to or worse than those observed in obese MASLD [100,101]. These observations highlight the need for more refined patient stratification integrating metabolic profiling, non-invasive fibrosis staging, and, when appropriate, genetic risk markers. In this context, future clinical algorithms are likely to incorporate multidimensional profiling to identify those patients most likely to benefit from incretin-based therapies versus alternative or complementary pharmacological approaches [100,101,102]. Another key issue concerns the positioning of incretin-based therapies relative to resmetirom, the first approved pharmacological agent for MASH with fibrosis. These therapeutic strategies target partially distinct but complementary aspects of disease biology. Resmetirom acts primarily as a liver-directed therapy through selective thyroid hormone receptor-β activation, improving hepatic lipid metabolism and reducing lipotoxicity. In contrast, GLP-1 receptor agonists and next-generation incretin co-agonists exert broader systemic metabolic effects, including weight loss, improvement of insulin resistance, reduction in systemic inflammation, and favorable modulation of cardiometabolic risk [103,104,105]. The therapeutic landscape is further evolving with the recent expansion of incretin-based therapies in MASH, including updates in clinical practice guidance incorporating semaglutide as a therapeutic option in selected patients with metabolic dysfunction and liver disease. This development represents a paradigm shift, as incretin-based drugs are no longer confined to metabolic indications but are increasingly entering the domain of liver-directed therapy. Future research should focus on head-to-head comparisons, real-world effectiveness, and combination treatment strategies [103]. Equally important for future implementation is the management of adverse events and treatment adherence. Gastrointestinal adverse effects—particularly nausea, vomiting, diarrhea, and constipation—remain the most common limitations of GLP-1-based therapies and are a leading cause of early treatment discontinuation. Recent evidence emphasizes the importance of gradual dose escalation, patient education, dietary counseling, and structured follow-up to improve tolerability and long-term persistence [104,105]. Although serious adverse events are generally uncommon, discontinuation rates remain higher than with placebo, underscoring the need for proactive management strategies in clinical practice [104]. From a broader perspective, the future management of MASLD will likely rely on the development of multidisciplinary care models, integrating hepatologists, endocrinologists, obesity specialists, nutritionists, and primary care physicians. Such an approach is essential not only for optimizing therapeutic selection and sequencing, but also for improving adherence and long-term outcomes. In parallel, the increasing use of non-invasive tests for fibrosis assessment and disease monitoring will play a central role in guiding therapeutic decisions, although their interpretation may be influenced by metabolic factors such as obesity and diabetes [105]. Finally, a major future goal is to transition from therapies that primarily improve surrogate endpoints (e.g., liver enzymes or hepatic fat content) to truly disease-modifying strategies capable of altering the natural history of MASLD. Achieving this objective will require long-term studies with histological and clinical endpoints, as well as a deeper understanding of disease heterogeneity and treatment response. In this evolving scenario, incretin-based therapies are likely to play a central role in personalized and outcome-oriented treatment strategies.

## 10. Conclusions

MASLD is one of the fastest-growing chronic liver diseases worldwide and is closely linked to the global epidemics of obesity, insulin resistance, and type 2 diabetes mellitus. Its rising prevalence, together with its potential progression to advanced fibrosis, cirrhosis, and hepatocellular carcinoma, represents a major and evolving public health challenge. Over the past decade, incretin-based therapies have emerged as a promising pharmacological approach capable of targeting the metabolic dysfunction that underlies hepatic steatosis and disease progression. Clinical trials and experimental studies indicate that incretin-based therapies can improve several metabolic and liver-related parameters, including body weight, insulin sensitivity, aminotransferase levels, and hepatic fat content. However, the strength of evidence differs substantially across drug classes and endpoints. For selective GLP-1 receptor agonists, semaglutide currently provides the most advanced histological evidence in MASH, whereas tirzepatide has generated strong phase 2 biopsy-based data as a dual GIP/GLP-1 receptor agonist. In contrast, GLP-1/glucagon receptor co-agonists and triple agonists have shown impressive anti-steatotic and metabolic effects, but their evidence remains more dependent on MRI-PDFF, liver enzymes, and other surrogate outcomes. Therefore, while these agents collectively support the concept of metabolically driven liver therapy, their clinical interpretation must remain anchored to the type of endpoint assessed. More recently, triple agonists targeting GLP-1, GIP, and glucagon receptors have demonstrated unprecedented effects on body weight and hepatic steatosis in early-phase clinical trials, suggesting that multi-receptor incretin modulation may represent a major therapeutic advance in MASLD. These agents have the potential to simultaneously address multiple pathogenic pathways, including insulin resistance, adipose tissue dysfunction, hepatic lipid accumulation, and energy expenditure, thereby offering a more comprehensive metabolic correction. Taken together, incretin-based therapies appear to exert hepatic effects through a continuum between indirect metabolic improvements and direct intrahepatic mechanisms. GLP-1 receptor agonists appear to act predominantly through weight loss-mediated pathways, whereas dual agonists, particularly those incorporating glucagon receptor activity, may provide additional direct hepatic metabolic modulation. Triple agonists represent the most advanced form of this paradigm, integrating profound systemic metabolic effects with potential intrahepatic actions, although the relative contribution of these mechanisms remains difficult to disentangle in clinical settings. This conceptual framework highlights the need for cautious interpretation of current clinical data, as many observed hepatic benefits may largely reflect global metabolic improvements rather than direct hepatocellular targeting. Despite these encouraging findings, several key questions remain unresolved. Most available studies rely on surrogate endpoints such as liver enzymes or imaging-based measures of steatosis, while robust data on histological outcomes—particularly fibrosis regression—are still limited. Long-term, large-scale randomized controlled trials are therefore needed to determine whether incretin-based therapies can truly modify the natural history of MASLD and prevent clinically relevant outcomes such as cirrhosis, hepatic decompensation, hepatocellular carcinoma, and liver-related mortality. In addition, further research is required to disentangle the relative contribution of weight loss versus direct hepatic effects, as well as to better understand the role of the gut–liver axis, the intestinal microbiome, and systemic inflammation in mediating therapeutic responses. Another critical area is the identification of patient subgroups most likely to benefit from these therapies, particularly in the context of emerging evidence supporting the heterogeneity of MASLD, including differences between obese and non-obese phenotypes. From a clinical perspective, the future management of MASLD will likely involve integrated and personalized strategies, combining lifestyle interventions, pharmacological therapies targeting metabolic derangement, and careful risk stratification based on fibrosis stage and cardiometabolic profile. Incretin-based therapies are expected to play a central role in this framework, especially in patients with coexisting obesity and type 2 diabetes, but their optimal positioning either as monotherapy or in combination with liver-directed agents such as resmetirom remains to be fully defined. Finally, the successful implementation of these therapies in real-world practice will require attention not only to efficacy but also to tolerability, adherence, cost, and accessibility, as well as the development of multidisciplinary care models involving hepatologists, endocrinologists, and primary care physicians. In conclusion, advances in incretin pharmacology and a deeper understanding of MASLD pathophysiology are reshaping the therapeutic landscape of this disease. While current evidence is highly promising, the ultimate goal will be to translate these developments into disease-modifying strategies capable of halting or reversing liver damage and improving long-term clinical outcomes.

## Figures and Tables

**Table 1:** Summary of the studies evaluating the effects of GLP-1 receptor agonists on liver disease.

Year	First Author	Study Design	Sample Size	Evaluated Drugs	Results	Key Points
2013	Fan H. [58]	Prospective randomized trial	117 patients with T2DM and NAFLD patients	Exenatide 5 µg twice daily (weeks 1–4) → 10 µg twice daily (weeks 5–12) vs. metformin 0.5 g twice daily → titrated up to 2.0 g daily (depending on blood glucose levels)	↓ liver enzymes (AST/ALT) and metabolic parameters (blood glucose and body weight)	Surrogate endpoints (liver enzymes). Short duration (12 W). Moderate strength of evidence.
2015	Eguchi Y. [59]	Prospective uncontrolled	19 patients with NASH	Liraglutide 0.9 mg/day for 24 weeks → treatment may be continued if effective and well tolerated	↓ liver enzymes (AST/ALT), metabolic parameters (blood glucose and body weight) and histological improvement (subset biopsy)	Histological and surrogate endpoints (liver enzymes). Small sample. No control group. Low-moderate strength of evidence.
2017	Feng W. [60]	Randomized clinical trial	87 patients with T2DM and NAFLD	Liraglutide 0.6 mg/day (week 1) → 1.2 mg/day (week 2) → 1.8 mg/day (≥week 3) vs. Gliclazide 30 mg/day → titrated up to 120 mg/day (target FPG < 7.0 mmol/L) vs. Metformin 250 mg tid (week 1) → 500 mg tid (week 2) → 1000 mg bid (≥week 3)	↓ liver enzymes (ALT) and intrahepatic fat (IHF)	Surrogate endpoints (liver enzymes and imaging). Moderate strength of evidence.
2017	Seko Y. [61]	Retrospective study	15 biopsy-proven NAFLD and T2DM patients	Dulaglutide 0.75 mg/week for 12 weeks	↓ liver enzymes (AST/ALT), metabolic parameters (hemoglobin A1c and body weight) and liver stiffness	Surrogate endpoints (liver enzymes and liver stiffness). Very small sample. Low strength of evidence.
2018	Cusi. [62]	Placebo- controlled clinical trial	1499 T2DM and NAFLD patients	Dulaglutide 1.5 mg/week (post hoc analysis of AWARD trials) vs. placebo	↓ liver enzymes (AST/ALT) and gamma-glutamyl transpeptidase levels (GGT)	Surrogate endpoints (liver enzymes). Post hoc analysis. Moderate strength of evidence.
2021	Newsome [49]	Prospective double-blind	320 obese/overweight NASH patients	Semaglutide 0.1/0.2/0.4 mg/day (s.c.) vs. placebo, 72 weeks vs. placebo	↑ NASH resolution	Histological endpoint. No fibrosis improvement. High strength of evidence.

**Table 2:** Summary of the studies of Dual GLP 1 + GIP agonist’s effect on MASLD.

Year	First Author	Study Design	Sample Size	Evaluated Drugs	Results	Key Points
2018	Coskun T. [63]	Translational pharmacology study	Preclinical models and early clinical pharmacology data	Tirzepatide (LY3298176)	↓ metabolic parameters (blood glucose and body weight)	Preclinical study. Animal model. No clinical liver enpoints. Low strength of evidence.
2020	Willard F.S. [64]	Mechanistic pharmacology study	Cellular and receptor signaling experiments	Tirzepatide	Biased receptor signaling (GIP/GLP-1)	Mechanistic study. No clinical endpoints. Low strength of evidence.
2022	Gastaldelli A. [68]	Phase 3 randomized clinical trial substudy (SURPASS-3 MRI)	296 patients with type 2 diabetes	Tirzepatide 5, 10, or 15 mg once weekly vs. insulin degludec	↓ liver fat (MRI-PDFF) and visceral/subcutaneous adipose tissue	Surrogate endpoints (liver fat by MRI-PDFF). Moderate strength of evidence.
2024	Jeong B.K. [71]	Preclinical experimental study	Mouse model of MASLD progressing to hepatocellular carcinoma	Tirzepatide (experimental dosing in animal model)	Improvement in steatosis, fibrosis and tumor burden (histology in animals)	Histological endpoint in animal model. Limited translational relevance. Low strength of evidence.
2024	Loomba R. [69]	Phase 2 randomized clinical trial (SYNERGY- NASH)	190 patients with MASH and liver fibrosis	Tirzepatide 5, 10, or 15 mg weekly	↑ MASH resolution without fibrosis worsening	Histological endpoint. High strength of evidence.
2025	Liang J. [66]	Preclinical multi-omics mechanistic study	Mouse model	Tirzepatide (experimental dosing)	Improvement in lipid metabolism and mitochondrial function (metabolomics, lipidomics and proteomics analyses)	Preclinical study. Animal model. No clinical endpoints. Low strength of evidence.
2025	Hu W. [67]	Preclinical animal study	Diabetic mouse model induced by high-fat diet and streptozotocin	Tirzepatide (experimental dosing)	Improvement in hepatic steatosis and metabolic parameters. Regulation of gut microbiota composition and bile-acid metabolism.	Preclinical study. Animal data. Low strength of evidence.
2025	Li Y. [65]	Preclinical animal and cellular study	Mouse MASLD model + HepG2 cells	Tirzepatide 0.25 mg/kg weekly	↓ hepatic lipid accumulation (cellular and animal endpoints)	Preclinical study. No human validation. Low strength of evidence.
2026	Tekin Uzman D. [70]	Clinical observational study	Patients with MASLD and type 2 diabetes (small clinical cohort)	Tirzepatide (clinical dosing)	↓ liver biomarkers (AST/ALT/GGT), non-invasive fibrosis indices (FIB-4 index and APRI (AST to Platelet Ratio Index), and metabolic parameters (blood glucose, serum lipid profile and body weight)	Surrogate endpoints (liver enzymes and fibrosis scores). Small sample. Moderate strength of evidence.

**Table 3:** Summary of the studies of Dual GLP 1 + glucagon agonist’s effect on MASLD.

Year	First Author	Study Design	Sample Size	Evaluated Drugs	Results	Key Points
2021	Nahra R. [75]	Phase 2b randomized clinical trial	834 patients with overweight/obesity and T2DM	Cotadutide 100–300 µg daily (subcutaneous)	↓ liver biomarkers (AST/ALT/GGT), non-invasive fibrosis indices (FIB-4 index and NAFLD fibrosis score) and metabolic parameters (hemoglobin A1c, blood glucose, serum lipid profile and body weight)	Surrogate endpoints (liver enzymes and fibrosis scores). Moderate strength of evidence.
2023	Parker V.E.R. [76]	Clinical mechanistic study	50 participants with overweight/obesity and T2DM	Cotadutide (dose escalation up to ~300 µg/day)	Improvement in liver metabolism by increasing hepatic glycogenolysis and reducing liver fat.	Mechanistic study. Low strength of evidence.
2023	Romero- Gòmez M. [77]	Phase 2a randomized active-comparator trial	145 patients with NAFLD	Efinopegdutide 10 mg once weekly vs. semaglutide 1 mg weekly	↓ liver fat (MRI-PDFF)	Surrogate endpoints (liver fat by MRI-PDFF). Moderate strength of evidence.
2024	Shankar S.S. [74]	Randomized clinical trial in biopsy-proven MASH	100 patients with non-cirrhotic MASH and fibrosis	Cotadutide (dose escalation up to ~300 µg/day)	↓ liver enzymes (AST/ALT) and liver fat (MRI-PDFF). Improvements in metabolic parameters and exploratory histological signals	Surrogate endpoints (liver enzymes and liver fat by MRI-PDFF) and exploratory histological endpoint. Early phase. Limited histological robustness. Moderate strength of evidence.
2024	Harrison S.A. [79]	Phase 2 randomized double-blind placebo-controlled trial	94 patients with MASLD	Pemvidutide 1.2–2.4 mg weekly	↓ liver enzymes (ALT) and liver fat (MRI-PDFF).	Surrogate endpoints (liver enzymes and liver fat by MRI-PDFF). Moderate strength of evidence.
Ongoing (Clinical trial)	MK-6024-013 investigators [78]	Phase 2 clinical trial (ongoing)	Not yet published	Efinopegdutide (dose under investigation)	Histological resolution of NASH without worsening fibrosis under evaluation	Histological endpoint (planned).

**Table 4:** Summary of the studies of triple agonist’s effect on MASLD.

Year	First Author	Study Design	Sample Size	Evaluated Drugs	Results	Key Points
2024	Sanyal A.J. [84]	Phase 2a randomized clinical trial	98 patients with MASLD	Retatrutide 2–12 mg once weekly (dose escalation)	↓ liver enzymes (AST/ALT) and liver fat (MRI-PDFF)	Surrogate endpoints (liver enzymes and liver fat by MRI-PDFF). Moderate strength of evidence.
2023	Julio Rosenstock [85]	Phase 2, randomized, double-blind, placebo- and active-controlled, parallel-group clinical trial	~281 adults with T2DM	Once-weekly subcutaneous administration of retatrutide (multi-dose escalation arms) versus placebo and active comparator (dulaglutide)	Improvement in glycemic control and weight loss	Surrogate endpoints (metabolic). No liver-specific outcomes. Moderate strength of evidence.

## Data Availability

No new data were created or analyzed in this study. Data sharing is not applicable to this article.

## Abbreviations

The following abbreviations are used in this manuscript:

AST	aspartate aminotransferase
ALT	alanine aminotransferase
DPP-4	dipeptidyl peptidase-4
FFAs	free fatty acids
GCG	glucagon
GIP	glucose-dependent insulinotropic polypeptide
GLP-1	glucagon-like peptide-1
GLP-1 RAs	glucagon-like peptide-1 receptor agonists
MASH	metabolic dysfunction-associated steatohepatitis
MASLD	metabolic dysfunction-associated steatotic liver disease
NAFLD	non-alcoholic fatty liver disease
T2DM	type 2 diabetes mellitus
THR-β	thyroid hormone receptor beta
